# Plasma GFAP outperforms CSF GFAP in detecting amyloid pathology and is associated with increased risk of clinical progression in early Alzheimer’s disease

**DOI:** 10.1016/j.tjpad.2026.100544

**Published:** 2026-03-28

**Authors:** Arda C. Cetindag, Carola G. Schipke, Hermann Esselmann, Niels Kruse, Jens Wiltfang, Anja Schneider, Klaus Fliessbach, Carolin Miklitz, Franziska Maier, Katharina Buerger, Daniel Janowitz, Michael Ewers, Sophia Stöcklein, Robert Perneczky, Boris-Stephan Rauchmann, Carolin Kurz, Stefan Teipel, Ingo Kilimann, Doreen Goerss, Christoph Laske, Sebastian Sodenkamp, Elham Najafpour, Michael Wagner, Sandra Roeske, Ingo Frommann, Melina Stark, Frederic Brosseron, Alfredo Ramirez, Luca Kleineidam, Josef Priller, Eike Jakob Spruth, Maria Gemenetzi, Slawek Altenstein, Emrah Düzel, Wenzel Glanz, Enise I. Incesoy, Michaela Butryn, Chris Bauer, Frank Jessen, Oliver Peters

**Affiliations:** a Charité Universitätsmedizin Berlin, Department of Psychiatry and Neurosciences, Hindenburgdamm 30, 12203 Berlin, Germany; b German Center for Neurodegenerative Diseases (DZNE), Berlin, Germany; c GLG Werner Forßmann Hospital, Department of Radiology and Neuroradiology, Rudolf-Breitscheid-Straße 100, 16225 Eberswalde, Germany; d University Medical Center Göttingen (UMG), Department of Psychiatry and Psychotherapy, Von-Siebold-Straße 5, 37075 Goettingen, Germany; e University Medical Center Göttingen (UMG), Department of Neuropathology, Robert-Koch-Straße 40, 37075 Goettingen, Germany; f German Center for Neurodegenerative Diseases (DZNE), Von-Siebold-Straße 3a, 37075 Goettingen, Germany; g Department of Old Age Psychiatry and Cognitive Disorders, University Hospital Bonn and University of Bonn, Bonn, Germany; h German Center for Neurodegenerative Diseases (DZNE), Bonn, Venusberg-Campus 1, 53127 Bonn, Germany; i Department of Psychiatry, University of Cologne, Medical Faculty, Kerpener Strasse 62, 50924 Cologne, Germany; j Institute for Stroke and Dementia Research (ISD), University Hospital, LMU Munich, Feodor-Lynen-Strasse 17, 81377 Munich, Germany; k German Center for Neurodegenerative Diseases (DZNE, Munich), Feodor-Lynen-Strasse 17, 81377 Munich, Germany; l Department of Radiology, University Hospital, LMU Munich, Munich, Germany; m Department of Psychiatry and Psychotherapy, LMU Hospital, LMU Munich, Germany; n Munich Cluster for Systems Neurology (SyNergy), Munich, Germany; o Epidemiology of Aging (AGE) Research Unit, School of Public Health, Imperial College London, London, UK; p Division of Neuroscience, University of Sheffield, Sheffield, UK; q Department of Neuroradiology, LMU Hospital, LMU Munich, Germany; r Department of Psychosomatic Medicine, Rostock University Medical Center, Gehlsheimer Str. 20, 18147 Rostock; s German Center for Neurodegenerative Diseases (DZNE), Rostock, Germany; t Section for Dementia Research, Hertie Institute for Clinical Brain Research and Department of Psychiatry and Psychotherapy, University of Tübingen, Tübingen, Germany; u Department of Psychiatry and Psychotherapy, University of Tübingen, Tübingen, Germany; v German Center for Neurodegenerative Diseases (DZNE), Tübingen, Germany; w Department for Cognitive Disorders and Old Age Psychiatry, University Hospital Bonn, Bonn, Germany; x Excellence Cluster on Cellular Stress Responses in Aging-Associated Diseases (CECAD), University of Cologne, Joseph-Stelzmann-Strasse 26, 50931 Köln, Germany; y Division of Neurogenetics and Molecular Psychiatry, Department of Psychiatry and Psychotherapy, Faculty of Medicine and University Hospital Cologne, University of Cologne, Cologne, Germany; z Department of Psychiatry & Glenn Biggs Institute for Alzheimer’s and Neurodegenerative Diseases, San Antonio, TX, USA; aa Department of Psychiatry and Psychotherapy, Charité, Charitéplatz 1, 10117 Berlin, Germany; bb University of Edinburgh and UK DRI, Edinburgh, UK; cc Department of Psychiatry and Psychotherapy, School of Medicine and Health, Technical University of Munich, and German Center for Mental Health (DZPG), Munich, Germany; dd German Center for Neurodegenerative Diseases (DZNE), Magdeburg, Germany; ee Institute of Cognitive Neurology and Dementia Research (IKND), Otto-von-Guericke University, Magdeburg, Germany; ff Neurosciences and Signaling Group, Institute of Biomedicine (iBiMED), Department of Medical Sciences, University of Aveiro, 3810-193, Aveiro, Portugal; gg MicroDiscovery GmbH, Berlin, Germany

**Keywords:** Alzheimer’s disease, Mild cognitive impairment, Subjective memory decline, Glial fibrillary acidic protein (GFAP), Cerebrospinal fluid (CSF), Blood biomarkers of Alzheimer’s disease

## Abstract

**Background:**

Early and accurate detection of Alzheimer’s disease (AD) is essential for timely intervention and development of disease-modifying treatments. The DZNE-Longitudinal Cognitive Impairment and Dementia Study (DELCODE) provides a deeply phenotyped cohort covering preclinical and early clinical stages, including subjective cognitive decline (SCD) and mild cognitive impairment (MCI). Astrocyte reactivity and its biomarkers, particularly glial fibrillary acidic protein (GFAP), have gained increasing attention in AD research; however, the relationship between GFAP and amyloid in early disease, as well as its potential prognostic value beyond its association with amyloid status, remains insufficiently understood.

**Objectives:**

To evaluate the performance of CSF and plasma GFAP across early disease stages, compare these measures according to amyloid status, and assess the prognostic value of GFAP for clinical progression across diagnostic stages during longitudinal follow-up.

**Setting:**

This study used data from the multicenter DELCODE cohort in Germany, including participants with available plasma and/or CSF samples and standardized clinical, cognitive, imaging, and biomarker assessments.

**Measurements:**

GFAP concentrations in plasma and CSF were quantified using validated immunoassay platforms. Standard CSF AD biomarkers and ApoE genotype were measured using established assays. Amyloid status was defined by the CSF Aβ42/40 ratio. Longitudinal follow-up occurred annually for up to ∼10 years, with clinical conversion determined according to NIA-AA criteria.

**Results:**

Plasma and CSF GFAP increased across the AD continuum, with higher levels in MCI and AD (p < 0.001). Plasma GFAP showed a stronger association with amyloid status than CSF GFAP across all groups. In MCI, plasma GFAP combined with age and ApoE4 yielded an AUC of 0.87. Elevated plasma GFAP predicted increased risk of conversion to MCI (HR = 2.19, p < 0.001; adjusted HR = 1.70, p = 0.0056) and AD dementia (HR = 3.5; adjusted HR = 2.49 both p < 0.001).

**Conclusion:**

Plasma GFAP is a sensitive, minimally invasive biomarker with diagnostic relevance for amyloid detection and prognostic relevance for clinical progression in early AD.

## 1 Introduction

Alzheimer’s disease (AD) is the most common cause of dementia globally and remains one of the leading causes of death and disability [1]. The amyloid cascade hypothesis continues to be a cornerstone in Alzheimer's disease (AD) research, driving numerous clinical investigations. Recently, two anti-amyloid monoclonal antibodies have received conditional FDA approval, marking a significant shift toward disease-modifying therapies aimed at individuals with mild cognitive impairment (MCI) or early AD dementia. These recent advancements, supported by updated diagnostic frameworks and therapeutic breakthroughs, underscore the urgent need for robust early detection tools—enabling intervention before irreversible neurodegeneration occurs and to meet the growing demand in cognitive care [2,3].

One hypothesis explaining the buildup of amyloid in the preclinical stage involves astrocyte reactivation in the brain [4]. In neurodegenerative diseases such as AD, it is well-established that astrocytes undergo various morphological and functional changes collectively referred to as reactive astrocytes [4]. Although the mechanisms underlying these processes remain poorly understood, the maturation of amyloid plaques has been linked to astrocyte reactivation [[4], [5], [6], [7]]. If early astrocyte reactivation plays a critical role in amyloid accumulation, focusing clinical research on this phenomenon could pave the way for new treatments targeting this dysfunction and enhance early detection efforts in the future.

At the same time, reactive astrocytes overexpress various proteins, such GFAP, an intermediate filament (IF) component of the astrocytic cytoskeleton, which plays key roles in neuronal regulation [4]. A possible relationship between astrocyte reactivation and amyloid accumulation has been reported in some of the old studies [[4], [5], [6], [7]]. Previous studies have demonstrated significantly higher GFAP levels in the cerebrospinal fluid (CSF) of AD patients compared to healthy controls [4,[8], [9], [10]]. Similarly, elevated serum/plasma GFAP levels have been consistently reported in Alzheimer's disease (AD) patients [[11], [12], [13], [14], [15], [16], [17], [18]] However, GFAP levels in individuals with mild cognitive impairment (MCI) or subjective cognitive decline (SCD) remain largely unexplored [17,[19], [20], [21]].

A recent study revealed that GFAP levels were lower in the normal biomarker group compared to those with prodromal AD and Alzheimer’s dementia [22]. Another study demonstrated that serum GFAP levels could serve as an indicator of Alzheimer’s pathology in patients with MCI [20]. Similarly, Cicognola et al. [16] demonstrated that plasma GFAP levels were significantly higher in Aβ-positive MCI patients compared to Aβ-negative ones, highlighting its potential as a biomarker to differentiate amyloid pathology in this early disease stage.

Furthermore, GFAP has recently attracted attention due to preliminary evidence showing its potential as a plasma biomarker that performs better than its CSF counterpart in detecting AD pathology [23]. Plasma GFAP more effectively differentiated Aβ-positive individuals from Aβ-negative ones compared to CSF GFAP (area under the curve for plasma GFAP, 0.69–0.86; for CSF GFAP, 0.59–0.76) [11]. These findings indicate that GFAP may be particularly associated with AD-related astrocytic activation and Aβ pathology, suggesting that it could serve as a promising blood biomarker for detecting and tracking disease processes [11].

However, GFAP has not yet been sufficiently validated as an early-stage biomarker across the Alzheimer’s disease spectrum, partly because existing studies often rely on modest sample sizes and lack long-term clinical follow-up. Consequently, little is known about the extent to which GFAP reflects disease processes in preclinical stages—such as subjective cognitive decline (SCD) and mild cognitive impairment (MCI)—or whether it provides information beyond amyloid pathology. In this study, we examined GFAP levels in plasma and cerebrospinal fluid (CSF) in participants of the DELCODE cohort and evaluated their association with amyloid status. Importantly, we also investigated the prognostic value of GFAP for future clinical progression, independent of amyloid, to assess its potential relevance for early disease stratification.

The DELCODE cohort provides deep phenotyping and longitudinal follow-up across healthy controls, relatives of Alzheimer’s patients, subjective cognitive decline (SCD), mild cognitive impairment (MCI), and Alzheimer’s disease (AD), allowing investigation of GFAP across early stages of the disease continuum and in individuals at increased familial risk. Given that SCD and MCI represent key pre-dementia at-risk states and central targets for early detection and prevention efforts, our analyses primarily focused on these groups. MCI due to AD is a well-established pre-dementia stage, while SCD reflects an even earlier at-risk state and has gained increasing attention as a promising window for AD prevention [24].

## 2 Methods

### 2.1 Study population

In this study, we utilized participants from the DELCODE cohort, part of the German Center for Neurodegenerative Diseases (DZNE), who had the required biological samples available. DELCODE is a multicenter, clinic-based observational longitudinal memory study. The study population included healthy control subjects (n=88), relatives of Alzheimer’s patients (n=44), patients with subjective cognitive decline (SCD, n=207), patients with mild cognitive impairment (MCI, n=112), and Alzheimer’s disease (AD, n=65) with available cerebrospinal fluid (CSF) samples. Additionally, plasma samples were available for healthy control subjects (n=191), relatives of Alzheimer’s patients (n=68), patients with SCD (n=391), MCI patients (n=170), and AD patients (n=108).

In the analysis where we compared amyloid-positive (A+) and amyloid-negative (A-) patients within diagnostic groups, patients were classified into amyloid-positive (A+) and amyloid-negative (A-) subgroups based on their cerebrospinal fluid (CSF) amyloid-beta 42/40 ratio. The ratio cut-off was 0.08, based on a data-driven approach of the DELCODE amyloid ratio data [25]. Only patients with both CSF and plasma results available were included. The final sample included healthy control subjects (n=59; A+: 12, A-: 47), patients with subjective cognitive decline (n=173; A+: 61, A-: 112), patients with mild cognitive impairment (n=95; A+: 57, A-: 38), and Alzheimer’s disease patients (n=55; A+: 51, A-: 4)

All patients underwent comprehensive clinical assessments at the respective memory centers before enrollment in the DELCODE study. These assessments included anamnesis, psychiatric and neurological examinations, neuropsychological testing, magnetic resonance imaging (MRI), lumbar puncture, and blood sampling. For a detailed description of the DELCODE study population, please refer to Jessen et al [24].

### 2.2 Definition of patient groups and cognitive assessment

The Consortium to Establish a Registry for Alzheimer`s Disease (CERAD) test battery was used to assess cognitive function before inclusion [24]. The SCD group was defined by the presence of subjectively reported memory decline with a test performance of better than -1.5 standard deviations (SD) of the normal range in all parts of the CERAD neuropsychological battery [24]. The MCI group was defined by a performance below -1.5 SD in the delayed recall trial of the CERAD word-list episodic memory tests. In the MCI group, only subjects with amnestic MCI were recruited [24]. Participants from both groups (SCD and MCI) fulfilled the current research criteria [24,[26], [27], [28]]. Additionally, participants with mild AD (Mini-Mental-Status-Examination (MMSE) ≥18) were included in the AD group, determined by National Institute on Aging and Alzheimer´s Association (NIA-AA) criteria [24,28,29]. The neuropsychological assessment battery at baseline and during annual follow-ups included the Alzheimer's Disease Assessment Scale (ADAScog 11/ 13), Clock Drawing and Clinical Dementia Rating (CDR). Cognitive tests at all memory clinics were rated by trained neuropsychologists. For detailed study design of the DELCODE cohort, see Jessen et al [24].

All participants underwent longitudinal clinical assessments approximately once per year. The maximum observation period was ∼10 years, and the median follow-up time was 4.98 years. Time-to-event was defined as the interval between the baseline visit and either the first visit at which conversion to MCI or AD was diagnosed, or the last available visit for censored individuals. Participants with MCI or dementia at baseline were excluded from the respective analyses. Conversion to MCI or AD during follow-up was defined according to current NIA-AA research criteria, including objective cognitive decline in at least one domain, associated functional impairment for dementia, and clinical adjudication by the responsible study physician.

### 2.3 Plasma analyses

Prior to analysis, samples were stored at -80°C. Approximately one hour before analysis, samples were thawed at room temperature and centrifuged for 10 min at 10.000 g. Plasma levels of GFAP were quantitated using the Simoa® GFAP Discovery Kit (PN 102,336, Quanterix, Billerica, MA, USA) on a SIMOA HD-X platform. Samples were transferred to a 96-well Quanterix® plate and duplicates were taken from a single well per sample. Each plate of samples tested included an eight point calibration curve, two controls, a plasma control pool and plasma samples. Controls, plasma pool and plasma samples were run using the 4x dilution protocol.

Plasma GFAP levels were analyzed in a total of 928 participants across five groups: healthy control subjects (n = 191), relatives of people with Alzheimer’s disease (n = 68), individuals with subjective cognitive decline (SCD) (n = 391), mild cognitive impairment (MCI) (n = 170), and Alzheimer’s disease (AD) patients (n = 108).

To ensure the quality and consistency of the plasma GFAP measurements, potential batch effects were assessed and corrected (Supplementary Figure 1). Initial analyses indicated variability between measurement batches, likely stemming from technical rather than biological differences. A batch correction was performed using an additive median adjustment on the logarithmic scale to normalize the data. After correction, batch variability was minimized, ensuring consistent and reliable data for subsequent statistical analyses.

**Fig. 1:**
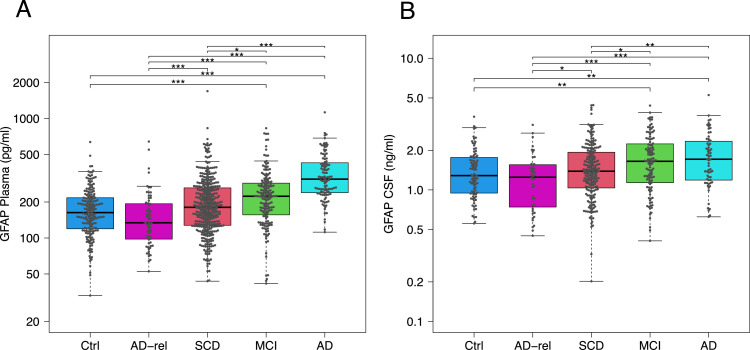
Biomarker Levels in Plasma and CSF across Different Groups. **(A)** Plasma GFAP (Glial Fibrillary Acidic Protein) levels (pg/ml) across subject groups, including healthy controls (Ctrl), relatives of Alzheimer’s disease patients (AD-rel), subjective cognitive decline (SCD), mild cognitive impairment (MCI), and Alzheimer’s disease (AD). Both the MCI and AD groups show significantly higher GFAP levels compared to controls (*p < 0.05, **p < 0.01, ***p < 0.001). **(B)** CSF GFAP (ng/ml) levels in the same groups. GFAP concentrations in both MCI and AD are also significantly higher than in controls (*p < 0.05, **p < 0.01, *p < 0.001).

**Fig. 2:**
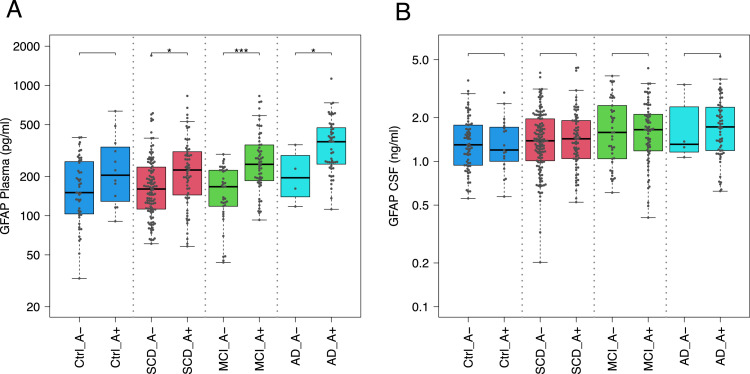
Biomarker Levels in Plasma and CSF Based on Amyloid-β Status. **(A)** Plasma GFAP levels (pg/ml) in the same groups. Plasma GFAP concentrations in SCD-A+, MCI-A+, and AD-A+ are significantly higher than in controls and their respective A− counterparts. **(B)** CSF GFAP (Glial Fibrillary Acidic Protein) levels (ng/ml) across subject groups categorized by amyloid-β negative (A−) and positive (A+) status, including healthy controls (Ctrl), subjective cognitive decline (SCD), mild cognitive impairment (MCI), and Alzheimer’s disease (AD). No significant differences were observed between A− and A+ groups in any category (p > 0.05) (*p < 0.05, **p < 0.01, ***p < 0.001).

**Fig. 3:**
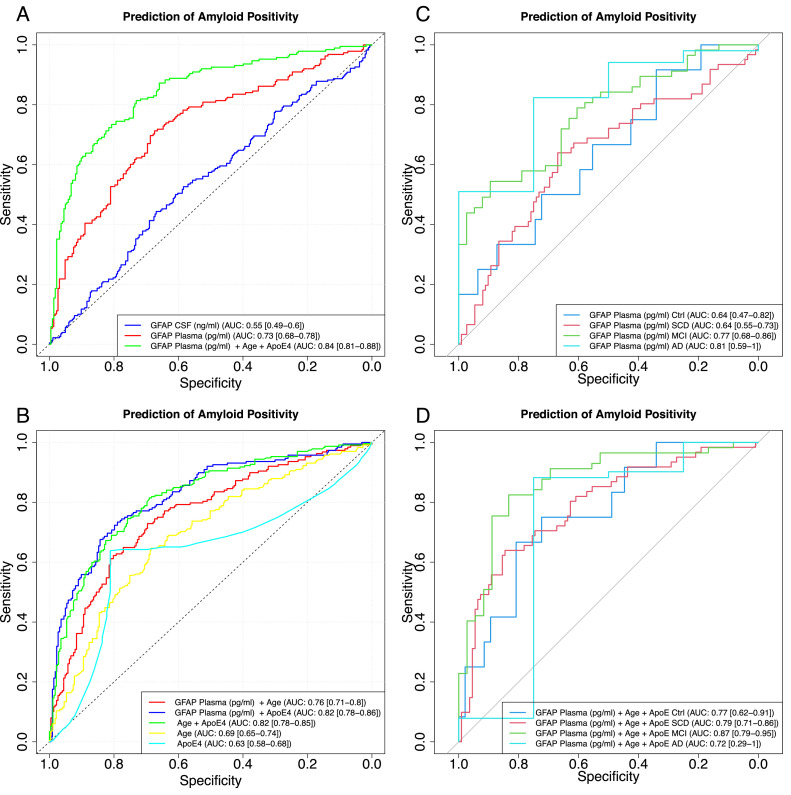
Receiver Operating Characteristic (ROC) Curves for prediction of amyloid positivity. **(A)** ROC curves comparing CSF GFAP, plasma GFAP alone, and plasma GFAP combined with age and ApoE4 in the total cohort. Plasma GFAP alone showed moderate discrimination (AUC: 0.73 [0.68–0.78]), whereas CSF GFAP showed lower performance (AUC: 0.55 [0.49–0.60]). The combined model including plasma GFAP, age, and ApoE4 demonstrated the highest classification accuracy (AUC: 0.84 [0.81–0.88]).**(B)** ROC curves comparing plasma GFAP + age (AUC: 0.76 [0.71–0.81]), plasma GFAP + ApoE4 (AUC: 0.82 [0.78–0.86]), age + ApoE4 (AUC: 0.82 [0.78–0.85]), age alone (AUC: 0.69 [0.65–0.74]), and ApoE4 alone (AUC: 0.63 [0.58–0.68]) for prediction of amyloid positivity.**(C)** ROC curves for plasma GFAP alone in individual diagnostic groups. Discriminatory performance varied across groups, with AUCs of 0.64 [0.47–0.82] for controls, 0.64 [0.55–0.73] for SCD, 0.77 [0.68–0.86] for MCI, and 0.81 [0.59–1.00] for AD.**(D)** ROC curves for plasma GFAP combined with age and ApoE4 within individual diagnostic groups. The combined model yielded AUCs of 0.77 [0.62–0.91] for controls, 0.79 [0.71–0.86] for SCD, 0.87 [0.79–0.95] for MCI, and 0.72 [0.29–1.00] for AD.

**Fig. 4:**
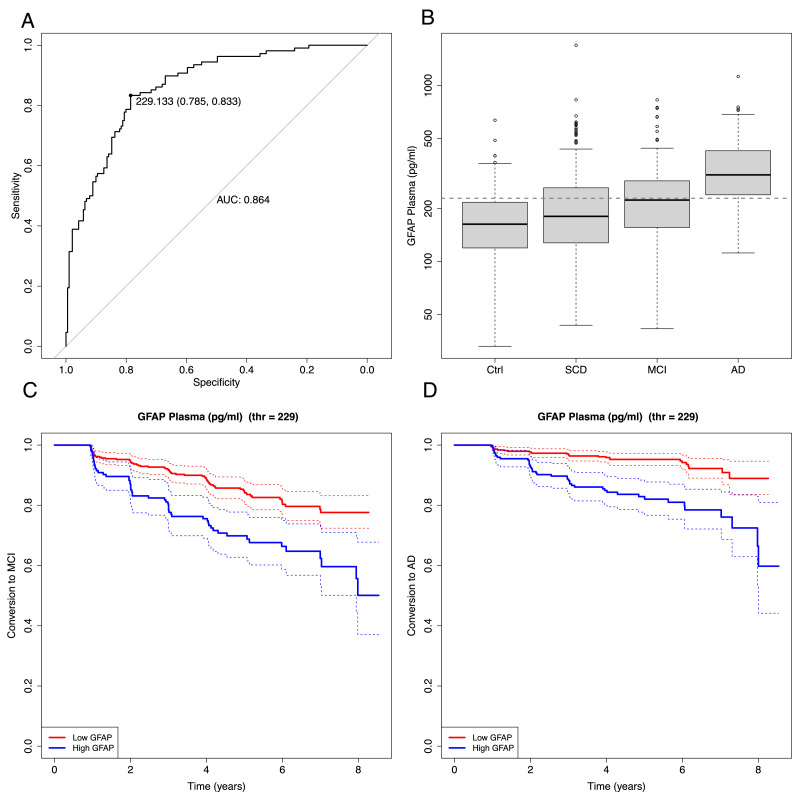
Plasma GFAP increases the amyloid-independent risk of clinical progression to MCI and dementia. **(A)** ROC curve of plasma GFAP differentiating AD from controls (AUC = 0.864). The optimal threshold (229 pg/ml), determined using the Youden index, yielded a sensitivity of 0.785 and a specificity of 0.833. **(B)** Distribution of plasma GFAP concentrations across diagnostic groups (Control, SCD, MCI, and AD). The dashed horizontal line indicates the ROC-derived optimal cutoff (229 pg/ml) used for subsequent survival analyses. **(C)** Kaplan–Meier curves for conversion to MCI stratified by plasma GFAP levels, showing faster conversion in the high-GFAP group (unadjusted HR = 2.19, 95 % CI: 1.53–3.14; adjusted HR = 1.70, 95 % CI: 1.17–2.47). **(D)** Kaplan–Meier curves for conversion to dementia, demonstrating a markedly increased progression risk in participants with high plasma GFAP (unadjusted HR = 3.5, 95 % CI: 2.17–5.65; adjusted HR = 2.49, 95 % CI: 1.52–4.08).

To investigate associations between GFAP levels and demographic as well as genetic variables, multivariate ANOVA models were performed including age, sex, years of education, and ApoE4 status. For plasma GFAP, significant associations were observed with age (p < 0.001), sex (p < 0.001), and ApoE4 status (p < 0.001), as well as a smaller association with years of education (p = 0.022). The association with ApoE4 is consistent with its established link to amyloid burden and downstream glial activation across the AD continuum. Sex and education were included as standard demographic covariates. Age correction was not applied to plasma GFAP in the group comparisons shown in Fig. 1, Fig. 2. Longitudinal data from our cohort showed inconsistent GFAP changes over time in controls independent of age. As amyloid levels increase with age in healthy individuals [30], and it remains unclear whether this directly drives astrocyte reactivation, we chose not to apply age correction in order to preserve biologically relevant variability. In Fig. 3, age and ApoE4 were included as covariates in the multivariate logistic regression models for amyloid prediction, such that reported effects are independent of these factors. In the longitudinal analyses (Fig. 4), hazard ratios are presented both unadjusted and adjusted for age, sex, and ApoE4, allowing assessment of the independent prognostic effect of plasma GFAP.

### 2.4 CSF analyses

516 patients were selected from the DELCODE cohort based on the availability of the required samples. GFAP concentrations were quantified using enzyme-linked immunosorbent assay (ELISA) kits (IBL-International, Hamburg, Germany) across all groups: relatives of people with Alzheimer’s disease (n = 44), healthy controls (n = 88), SCD (n = 207), MCI (n = 112), and AD patients (n = 65). CSF samples from these subjects were analyzed in two separate time periods, 2017 and 2020. All CSF GFAP measurements were performed in duplicate, consistent with the plasma analyses. To control for potential plate effects, we assessed whether significant differences existed between plates. No plate effects were observed (F-test, p ≈ 0.05). Additionally, there were no notable differences in the proportion of AD stages across the different plates. To investigate associations between CSF GFAP levels and demographic as well as genetic variables, multivariate ANOVA models were performed including age, sex, years of education, and ApoE4 status. CSF GFAP was significantly associated with age (p < 0.001) and sex (p = 0.030), whereas no significant associations were observed for ApoE4 status (p = 0.239) or years of education (p = 0.116). Within the control group, CSF GFAP was not significantly associated with age (p = 0.40). Age correction was not applied to the group comparisons shown in Fig. 1, Fig. 2. For CSF GFAP, the main reason was the absence of a significant association with age within the control group (p = 0.40), indicating that CSF GFAP levels in controls are not simply driven by physiological aging. In addition, age is closely linked to amyloid accumulation and Alzheimer’s disease pathology [30]. Adjusting for age could therefore remove biologically meaningful disease-related variance. Applying the same approach as in the plasma analyses also ensured consistency across biofluids. In Fig. 3, age and ApoE4 were included as covariates in the multivariate logistic regression models, confirming that the reported associations reflect effects independent of these variables.

As part of the standard DELCODE cohort characterization**,** we also included established CSF AD biomarkers—Aβ42, the Aβ42/Aβ40 ratio, total tau (T-tau), and phosphorylated tau (P-tau-181). These biomarkers were quantified using commercially available kits in accordance with the manufacturers’ instructions: V-PLEX Aβ Peptide Panel 1 (6E10) Kit (K15200E) and V-PLEX Human Total Tau Kit (K151LAE) (Mesoscale Diagnostics LLC, Rockville, USA), as well as Innotest Phospho-Tau(181P) (81,581; Fujirebio Germany GmbH, Hannover, Germany).

### 2.5 ApoE genotyping

Apolipoprotein E (ApoE) genotyping status is available in our dataset for n= 1021 (>99 %), Single nucleotide polymorphisms (SNPs) defining the ε-2, ε-3, and ε-4 alleles of ApoE, were genotyped using a commercially available TaqMan® SNP Genotyping Assay (Thermo Fisher Scientific). Both SNP assays were amplified on genomic DNA using a Step One Plus Real-Time PCR System (ThermoFisher Scientific). Visual inspection of cluster formation was performed for each SNP before genotype data were further used to define ε-2, ε-3, and ε-4 alleles in each sample [24].

### 2.6 Statistical analysis

All statistical analyses were performed with R version 4.4.2. Due to plate effect of GFAP data in plasma, we performed a batch normalization only for GFAP plasma data (in CSF no batch effect visible). The batch effects were corrected by performing an additive median normalization of logarithmic scale (which corresponds to a linear multiplicative transformation of natural scale).

For comparing statistical properties of baseline variables between different diagnoses, we used Fisher's exact test for categorical variables (age and ApoE4) and f-test (with ANOVA) for numerical variables.

After we confirmed that our data is normally distributed (Shapiro-Wilk test – data not shown) and corrected the plate effect of GFAP plasma data, we performed pairwise independent *t*-tests to compare the different groups. A *p*-value < 0.05 was defined as significant. Due to the exploratory nature of this study, a correction for multiple testing was not applied. The effect size was calculated using Cohen’s d.

Single value ROC curves (incl. AUC and CIs) were calculated using the R package ‘pROC’. Plasma GFAP was dichotomized into ‘high’ vs. ‘low’ levels using a threshold of 229 pg/mL, derived from the Youden index of a ROC curve contrasting AD patients and cognitively unimpaired controls at baseline. For multivariate logistic regression we applied a leave-10 out cross validation in order to avoid overfitting.

Time-to-event analyses for conversion to MCI or AD were conducted using the R package ‘survival’. Kaplan–Meier survival curves were generated, and group differences were evaluated using the log-rank test. Cox proportional hazards models were used to estimate hazard ratios, using dichotomized GFAP group (high vs. low) as the predictor variable. To assess whether the effect of plasma GFAP was independent of demographic and genetic factors, additional multivariate Cox regression analyses were performed including age, sex, and ApoE4 status as covariates. Numbers at risk at each time point were extracted separately for MCI and dementia outcomes.

## 3 Results

### 3.1 Demographic markers differ between groups

Group differences in categorical variables were assessed using Fisher’s exact test, and continuous variables using F-tests.

The AD group had fewer years of education and a lower proportion of females compared to the other groups (both p ≤ 0.004). Age differed significantly across groups, with AD participants being oldest, followed by MCI and SCD, while controls were youngest (p < 0.001).

As expected, neuropsychometric performance differed significantly between groups, with the AD group showing the most pronounced impairment across cognitive measures, including lower PACC5 and Clock Drawing scores and higher ADAS-Cog-11/13 scores (all p < 0.001).

Consistent with previous DELCODE reports [25], CSF Aβ42 levels and the Aβ42/Aβ40 ratio were lowest in AD, followed by MCI and SCD, compared with controls. ApoE4 carriership also differed across groups in line with prior findings [25] (Table 1).

**Table 1:** Table with statistics for demographic markers, neuropsychometric scores and classical CSF biomarkers.

	**AD-relatives**			**Ctrl**			**SCD**			**MCI**			**AD**			***p*-value**
	**Mean**	**SD**	**N**	**Mean**	**SD**	**N**	**Mean**	**SD**	**N**	**Mean**	**SD**	**N**	**Mean**	**SD**	**N**	
**Education years**	14.59	2.74	79	14.71	2.65	221	14.92	2.97	425	14.06	3.14	186	12.88	3.01	118	6.18E-10(<0.001)
**Sex**∗	0.61		79	0.57		221	0.51		69	0.46		425	0.59		118	4.00E-03
**Age**	65.51	4.40	79	68,60	5.09	221	70.89	6.05	425	72.43	5.54	186	74.73	6.34	118	1.41E-33(<0.001)
**PACC5**	0.14	0.72	79	0.20	0.54	221	-0.11	0.67	420	-1.48	1.02	172	-3.70	1.25	61	4.80E-208 (<0.001)
**ADAS-Cog-11**	3.32	2.05	79	3.17	1.62	221	3.77	2.05	424	8.94	4.51	184	19.05	7.37	116	9.72E-244 (<0.001)
**ADAS-Cog-13**	5.81	4.03	79	5.29	2.95	221	6.91	3.73	424	16.12	6.65	184	29.58	8.73	116	2.26E-266 (<0.001)
**Clock-drawing test**	9.03	1.14	79	9.13	1.16	221	9.09	1.27	424	8.20	1.88	184	6.20	2.74	116	4.83E-63 (<0.001)
**CSF Ratio** **Aβ -42/Aβ-40**	0.10	0.02	44	0.10	0.02	89	0.09	0.03	207	0.07	0.03	111	0.05	0.02	65	6.67E-33 (<0001)
**CSF Aβ-42**	853.08	338.33	44	844.18	296.42	89	774.51	336.54	207	585.68	307.12	111	411.52	198.46	65	2.53E-21 (<0.001)
**CSF Total-Tau**	342.94	127.01	44	366.70	160.05	89	369.26	185.25	207	544.48	298.64	111	805.34	374.44	65	1.77E-36 (<0.001)
**CSF Phospho-Tau 181**	49.79	18.32	44	50.14	17.89	89	53.83	24.16	207	70.92	41.88	111	96.56	43.63	65	9.25E-24 (<0.001)
**APOE 4 carriership**∗∗	0.35		79	0.21		220	0.32		422	0.49		183	0.63		117	5.00E-04 (<0.001)

Baseline features of the total study population are presented in the Table. Ctrl, Control; SCD, subjective cognitive decline; MCI, mild cognitive impairment; AD, Alzheimer`s Disease; MMSE, mini mental status test; ADAS-Cog-11, Alzheimer's Disease assessment scale 11; ADAS-Cog-13, Alzheimer's Disease Assessment Scale 13; CDR-SOB, clinical dementia rating scale sum of boxes; CDR-Global, clinical dementia rating scale global score; ApoE, Apolipoprotein E. For categorical variables (age and ApoE4) p-values were calculated with Fisher's exact test. For numerical variables p-values were calculated using the f-test (see column pv, p value).

∗ For sex the ’mean’ column represents the percentage of females.

∗∗ For ApoE 4 carriership ’mean’ column represents the percentage of subjects carrying at least one ApoE4 gene.

### 3.2 GFAP levels in CSF and blood differed significantly among disease stages

GFAP levels in CSF were higher in more advanced disease stages, with means (ng/ml) as follows: Ctrl (M = 1.43), SCD (M = 1.54), MCI (M = 1.76), AD-rel (M = 1.27), and AD (M = 1.86) (Fig. 1B). Compared with controls, CSF GFAP levels were significantly higher in AD (p < 0.001, Cohen’s d = 0.58) and MCI patients (p < 0.001, Cohen’s d = 0.44). Both AD and MCI groups also showed higher CSF GFAP levels than the SCD group (AD vs. SCD: p < 0.01, Cohen’s d = 0.41; MCI vs. SCD: p < 0.05, Cohen’s d = 0.29). In addition, CSF GFAP levels in SCD were higher than in the AD-rel group, representing healthy individuals with familial risk of Alzheimer’s (Fig. 1B).

Plasma GFAP levels showed a similar pattern across disease stages, with means (pg/ml) of Ctrl (M = 193), SCD (M = 209), MCI (M = 230), and AD (M = 304) (Fig. 1A). Plasma GFAP levels were significantly higher in AD (p < 0.001, Cohen’s d = 1.45) and MCI patients (p < 0.001, Cohen’s d = 0.58) compared to controls. Both AD and MCI groups also showed higher plasma GFAP levels than the SCD group (AD vs. SCD: p < 0.001; MCI vs. SCD: p < 0.05). SCD patients also showed higher plasma GFAP levels than AD-rel individuals (p < 0.001, Cohen’s d = 0.39) (Fig. 1A).

### 3.3 GFAP levels in CSF and blood across amyloid(+) and amyloid(-) groups

To assess differences between amyloid-negative (A−) and amyloid-positive (A+) individuals, defined by a CSF Aβ42/40 ratio ≤ 0.08, GFAP levels were analyzed across disease stages.

In CSF, mean GFAP levels (ng/ml) were similar between A(−) and A(+) groups across all stages (AD: 1.76 vs. 1.86; Ctrl: 1.44 vs. 1.41; MCI: 1.80 vs. 1.74; SCD: 1.52 vs. 1.58), with no significant differences observed in AD (p = 0.867) or MCI (p = 0.737) (Fig. 2B).

In contrast, plasma GFAP levels differed significantly between A(+) and A(−) groups across disease stages. Mean concentrations (pg/ml) were higher in A(+) individuals in AD (379.0 vs. 215.0), controls (259.0 vs. 180.0), MCI (294.0 vs. 163.0), and SCD (256.0 vs. 202.0) (Fig. 2A). These differences were significant for AD (p < 0.05), MCI (p < 0.001), and SCD (p < 0.05).

### 3.4 Multivariate logistic regression analysis for A(+) prediction

After observing the association between amyloid (A) positivity and plasma GFAP levels across disease stages, we evaluated the ability of plasma GFAP to predict amyloid positivity using multivariate logistic regression models including non-invasive covariates.

Using leave-10-out cross-validation, the model combining plasma GFAP, age, and ApoE4 genotype achieved an AUC of 0.84 [0.81–0.88], with a sensitivity of 0.793 and a specificity of 0.843 (Fig. 3A). Plasma GFAP alone showed an AUC of 0.73 [0.68–0.78], while CSF GFAP alone showed an AUC of 0.55 [0.49–0.60] (Fig. 3A). Pairwise DeLong comparisons showed that the combined model (GFAP + age + ApoE4) differed significantly from plasma GFAP alone (p < 0.001) and from CSF GFAP (p < 0.0001). Plasma GFAP alone also differed significantly from CSF GFAP (p < 0.001).

To assess the contribution of individual covariates, additional models were calculated (Fig. 3B). Age alone yielded an AUC of 0.69 [0.65–0.74], and ApoE4 alone yielded an AUC of 0.63 [0.58–0.68]. The combination of age + ApoE4 reached an AUC of 0.82 [0.78–0.85]. Plasma GFAP combined with age resulted in an AUC of 0.76 [0.71–0.81], and plasma GFAP combined with ApoE4 yielded an AUC of 0.82 [0.78–0.86].

Fig. 3C presents ROC analyses for plasma GFAP alone within individual diagnostic groups. AUC values were 0.64 [0.47–0.82] for controls, 0.64 [0.55–0.73] for SCD, 0.77 [0.68–0.86] for MCI, and 0.81 [0.59–1.00] for AD.

Fig. 3D shows ROC analyses for plasma GFAP combined with age and ApoE4 within diagnostic groups. The AUC was 0.77 [0.62–0.91] for controls, 0.79 [0.71–0.86] for SCD, 0.87 [0.79–0.95] for MCI, and 0.72 [0.29–1.00] for AD.

### 3.5 GFAP threshold and cox regression analysis

In the previous analysis, we observed a strong association between high GFAP levels in blood and amyloid positivity. Building upon this finding, we aimed to investigate whether GFAP levels could predict the progression to MCI or AD regardless of amyloid status. For this purpose, we established a GFAP threshold based on the Youden point of a single-value ROC curve comparing AD and control groups (Fig. 4 A-B)

Using this threshold (229 pg/ml), GFAP levels were dichotomized into high and low groups. Cox regression analysis revealed that individuals with high GFAP levels had a significantly increased hazard ratio (HR) for conversion to MCI (HR = 2.19, 95 % CI: 1.53–3.14, p < 0.001) and dementia (HR = 3.5, 95 % CI: 2.17–5.65, p < 0.001) compared to those with low GFAP levels (Fig. 4C–D).The Kaplan–Meier analysis revealed a clear separation over time between the high- and low-GFAP groups. For MCI conversion, the cumulative survival probability decreased from 0.97 at year 1 to 0.70 at year 5 in the high-GFAP group, compared with 0.98 and 0.85 in the low-GFAP group. Similarly, for dementia conversion, the high-GFAP group declined from 0.996 at year 1 to 0.83 at year 5, while the low-GFAP group showed only a modest decline from 0.996 to 0.95 over the same period. These estimates reflect a substantially higher event accumulation in the high-GFAP group.

To account for potential confounding effects, we additionally performed multivariate Cox regression analyses including age, sex, and ApoE4 status as covariates. Even after adjustment, elevated plasma GFAP remained a significant predictor of conversion to MCI (adjusted HR = 1.70, 95 % CI: 1.17–2.47, p = 0.0056) and to dementia (adjusted HR = 2.49, 95 % CI: 1.52–4.08, p < 0.001), indicating that the prognostic effect of GFAP is independent of these demographic and genetic factors.

To facilitate interpretation of the survival curves, for the MCI endpoint, the risk set decreased from 412 to 196 individuals in the low-GFAP group and from 161 to 66 in the high-GFAP group over the first five years. For dementia, risk sets declined from 491 to 245 (low GFAP) and 225 to 98 (high GFAP), illustrating differential dropout and event patterns between groups. Detailed Kaplan–Meier statistics, including numbers at risk, event counts, censoring information, confidence intervals, and cumulative hazard estimates for each time point, are provided in Supplementary Tables 1A (conversion to MCI) and 1B (conversion to dementia). Together, these analyses consistently show higher conversion rates in individuals with elevated plasma GFAP levels across both MCI and dementia endpoints.

## 4 Discussion

Our findings suggest the considerable diagnostic and prognostic potential of GFAP as a biomarker for AD across different disease stages. By directly comparing CSF and plasma GFAP levels, we demonstrated that plasma GFAP represents a stronger and more robust biomarker for detecting amyloid pathology, even in earlier disease stages**.**

Consistent with previous studies [4,9,11,31], we observed a progressive increase in GFAP levels from control to MCI and AD patients, indicating an association between GFAP levels and disease severity among affected individuals. The significant differences in GFAP levels between MCI/AD groups and controls in both CSF and blood further support the notion that GFAP reflects progressive glial activation and neuronal injury during disease advancement [9,11,31,32].

Notably, when considering the AD-rel group as a secondary healthy control group, we found that SCD patients – often regarded as a pre-MCI stage – exhibited significantly higher GFAP levels in both CSF and blood. This finding suggests that GFAP levels may already be elevated in preclinical stages, supporting its potential as an early indicator of glial activation and neurodegeneration. Accordingly, blood-based measurements may offer a promising, minimally invasive approach for early detection. These results are further supported by other longitudinal findings showing that plasma GFAP increases over time in individuals with genetic risk for AD, even before cognitive decline becomes apparent [33]. Additionally, recent large-scale evidence corroborates this relationship, demonstrating that elevated plasma GFAP not only precedes cognitive symptoms but also predicts long-term risk of both AD and all-cause dementia in diverse populations [34]. Collectively, these findings strengthen the view of GFAP as an early-stage biomarker and contribute to filling existing gaps by adding novel insights into its diagnostic potential, especially in preclinical populations such as the SCD group. Future research should aim to replicate these findings in other large cohorts and further explore the mechanisms driving these differences.

Furthermore, we observed that in blood, GFAP levels demonstrated statistically significant differences between A (+) and A (-) groups across all disease stages, including SCD. This highlights the potential of blood-based GFAP as a useful biomarker for distinguishing amyloid status, even in very early and preclinical stages of AD. Specifically, significant differences were observed between SCD-A(+) and SCD-A(−) groups, as well as between MCI-A(+) and MCI-A(−) groups, underscoring the sensitivity of GFAP in blood to amyloid pathology. In contrast, in CSF, GFAP levels did not exhibit significant differences between amyloid-positive and amyloid-negative groups in any disease stage. This indicates that GFAP levels in CSF are less influenced by amyloid status in early stages. The absence of significant differences in CSF GFAP levels between A(+) and A(−) groups in early disease stages underscores a key limitation of CSF GFAP for detecting amyloid pathology, particularly in preclinical phases such as SCD, where amyloid deposition in CSF likely represents a late and complex process. In contrast, higher detectability of plasma GFAP may be explained by differential astroglial clearance mechanisms and enhanced stabilization of GFAP in the peripheral circulation by blood proteins..

In line with these observations, our findings show that the observed association between plasma GFAP and Aβ is consistent with its established role in AD pathology, where Aβ deposition in plaques triggers astrogliosis and the subsequent release of GFAP from astrocytes [35]. Findings from previous amyloid-PET imaging studies suggest that the progressive accumulation of Aβ in the brain inversely correlates with astrogliosis during the later stages of the disease [36,37]. Similarly, a recent cross-sectional study demonstrated a positive correlation between plasma GFAP levels and brain Aβ load in the earlier stages of the disease, as measured by amyloid-PET, while this relationship weakens in advanced stages [38]. Furthermore, Cicognola et al. demonstrated that plasma GFAP is strongly associated with amyloid pathology specifically in MCI patients, highlighting its potential as a biomarker for detecting early amyloid-related changes in this group [16].

Following our observation of the significant association between amyloid positivity and GFAP levels across disease stages, we evaluated the predictive performance of GFAP using ROC curve analyses. Consistent with previous findings [11,16] blood GFAP outperformed CSF GFAP in distinguishing between A (+) and A (-) individuals. Although the group definitions in these studies differ slightly from ours, they provide valuable context. Specifically, the AUC for GFAP in plasma was 0.73, compared to 0.56 for GFAP in CSF, underscoring the superior utility of blood GFAP as a marker for amyloid pathology.

To enhance the prediction of amyloid status, we incorporated additional variables into a multivariate logistic regression analysis. By combining plasma GFAP with age and ApoE genotype, the model achieved a significantly improved AUC of 0.84, with sensitivity and specificity values of 0.793 and 0.843, respectively. This represents a notable enhancement compared to GFAP alone (AUC = 0.73). The relatively high AUC observed for the age + ApoE4 model reflects the well-established role of both age and ApoE4 as major determinants of amyloid accumulation in Alzheimer’s disease.

To further enhance the prediction of amyloid status, we performed additional analyses by stratifying the groups into Ctrl, SCD, MCI, and AD. Among these, the best performance was observed in the MCI group, where combining blood GFAP with age and ApoE genotype yielded an AUC of 0.87 [95 % CI: 0.79–0.95]**.** This result slightly surpasses the findings of Cicagnola et al. 2021 [16], who reported an AUC of 0.86 for the MCI group in their ROC curve analysis. Despite differences in group definitions and cohort sizes between the two studies, the higher AUC observed in our study further supports the strength and potential of blood GFAP as a biomarker for amyloid pathology, particularly in the MCI stage. These findings highlight that while GFAP alone is a strong predictor of amyloid positivity, its integration with easily accessible, non-invasive factors such as age and genetic risk (ApoE) substantially strengthens its predictive power. Beyond its diagnostic performance, the central question arising from these findings is whether plasma GFAP provides prognostic information on clinical progression over time.

To address this question, building upon the observed association between blood GFAP levels and amyloid positivity, we investigated whether baseline plasma GFAP could predict subsequent clinical progression to MCI or AD using longitudinal follow-up data. By applying a GFAP threshold of 229 pg/ml, derived from the Youden point of a ROC curve comparing AD patients and cognitively unimpaired controls, individuals were stratified into high and low GFAP groups at baseline.

Using time-to-event analyses, elevated baseline GFAP levels were associated with a substantially increased risk of clinical conversion. Specifically, individuals with high GFAP levels showed an approximately twofold higher hazard for conversion to MCI (unadjusted HR = 2.19, 95 % CI: 1.53–3.14, p < 0.001; adjusted HR = 1.70, 95 % CI: 1.17–2.47, p = 0.0056) and a markedly increased risk of conversion to dementia (unadjusted HR = 3.50, 95 % CI: 2.17–5.65, p < 0.001; adjusted HR = 2.49, 95 % CI: 1.52–4.08, p < 0.001). Kaplan–Meier analyses consistently demonstrated an earlier and steeper decline in conversion-free survival among participants with high GFAP levels, supporting the robustness of this association over the follow-up period.

Taken together, these longitudinal findings extend the diagnostic relevance of plasma GFAP by demonstrating its prognostic value for clinical progression across diagnostic groups. Rather than reflecting amyloid pathology alone, elevated plasma GFAP appears to capture astroglial activation and broader neurodegenerative processes that precede and accompany clinical deterioration. This positions blood-based GFAP as a promising biomarker for identifying individuals at increased risk of rapid progression, particularly in early and pre-dementia stages of Alzheimer’s disease.

Consistent with this concept, previous studies have demonstrated that GFAP immunoreactivity in astrocytes increases with the duration of clinical illness in AD [39], and reactive astrocytes are frequently observed in post-mortem AD brain tissue, often in association with increased Aβ or Tau pathology [[5], [6], [7]]. Beyond structural changes, reactive astrocytes actively contribute to neuroinflammatory cascades through the release of cytokines, inflammatory mediators, nitric oxide, and reactive oxygen species, thereby promoting redox imbalance and neuronal vulnerability [4,40].

Despite these advances, the precise role of astrocytes within the amyloid cascade hypothesis and the triggers of β-amyloid accumulation remain incompletely understood [4]. If early astroglial activation contributes to amyloid accumulation during preclinical stages, biomarkers of astrocyte reactivity may hold particular clinical value for identifying individuals at increased risk before overt cognitive decline. In this framework, GFAP represents a promising link between mechanistic insights into astrocyte biology and the clinical need for early, minimally invasive biomarkers, warranting further investigation of its relationship with Aβ and Tau to better understand astrocyte reactivity in early disease stages and its interaction with other pathological processes.

Blood-based biomarkers such as the plasma Aβ42/40 ratio and pTau217 have demonstrated substantial potential for the early detection of Alzheimer’s disease pathology. In the DELCODE cohort, the plasma AβX-42/X-40 ratio achieved an AUC of approximately 0.81 for amyloid positivity, increasing to 0.87 when combined with ApoE ε4 status [41], while plasma pTau217 has shown high diagnostic accuracy for amyloid and tau pathology in independent cohorts (AUCs ranging from 0.92–0.96 for amyloid and 0.93–0.97 for tau) [42]. In our MCI subgroup, the combination of plasma GFAP with covariates yielded an AUC of 0.87, comparable to the performance reported for plasma Aβ in DELCODE. These findings indicate that plasma Aβ42/40 and pTau217 provide robust information for detecting amyloid and tau-related pathology.

While plasma Aβ measurement relies on enrichment-based protocols to quantify low-abundance peptides, plasma GFAP can be measured using direct ultrasensitive immunoassays (41) and reflects glial activation processes beyond core amyloid pathology. In this context, GFAP may offer additional value by capturing neuroinflammatory changes that are not fully reflected by amyloid- or tau-centered markers alone. Ongoing advances in fully automated immunoassay platforms further suggest that GFAP measurement may become increasingly accessible for broader clinical use.

Longitudinal blood-based biomarker studies in early AD remain limited. Beyond its association with amyloid status, our longitudinal analyses show that elevated plasma GFAP levels predict a higher risk of clinical conversion. Taken together, these findings support a multimarker approach in which GFAP complements established amyloid and tau biomarkers by providing additional insight into glial activation and clinical progression. Within the DELCODE consortium, plasma pTau and NfL datasets are currently being analyzed in separate projects, and a systematic comparison with plasma GFAP is planned in a forthcoming publication.

## 5 Conclusion

This study demonstrates that GFAP is not only a marker of amyloid-related pathology but also a biomarker associated with increased risk of clinical progression across diagnostic stages in Alzheimer’s disease. Using time-to-event analyses, elevated plasma GFAP was robustly associated with an increased risk of conversion to both MCI and dementia during longitudinal follow-up, even after adjustment for demographic and genetic risk factors.

Importantly, plasma GFAP consistently outperformed CSF GFAP in detecting amyloid positivity at early and preclinical stages, supporting its role as a minimally invasive and clinically feasible biomarker. Beyond its diagnostic utility, our findings suggest that plasma GFAP captures astrocyte-related processes that contribute to disease progression beyond classical amyloid and tau pathways.

Together, these results position plasma GFAP as a promising tool for risk stratification and longitudinal monitoring, particularly in early and preclinical populations such as SCD. Future studies should validate these findings in larger longitudinal cohorts and assess the added prognostic value of GFAP in combination with emerging plasma biomarkers to further refine early disease prediction.

### 5.1 Limitations in this study and suggestions for future studies

This study has several limitations. First, non-Alzheimer’s dementia types were not included; therefore, CSF and plasma GFAP levels could not be compared between early AD and other neurodegenerative diseases. Future studies should address the specificity of GFAP by including non-AD dementia cohorts.

Second, although SCD is commonly regarded as a pre-MCI stage, this group is inherently heterogeneous, as not all individuals progress to AD. Some may remain stable or develop other neurodegenerative conditions, which limits the interpretation of findings in this subgroup. Longitudinal studies focusing on astrocytic dysfunction in SCD are needed to clarify these trajectories.

Third, potential circadian influences on GFAP levels were not assessed, which may affect biomarker reproducibility. In addition, although age was included as a covariate, age-related biological effects on GFAP were not examined in detail and may influence generalizability, particularly in older populations.

Finally, the DELCODE cohort predominantly includes individuals of European ancestry, limiting applicability to other populations.

## Funding

DELCODE study is financed by the DZNE (Deutsches Zentrum für Neurodegenerative Erkrankungen, DZNE).

### Availability of data and materials

The datasets generated and/or analyzed during the current study are not publicly available due to local data storage but are available from the corresponding author upon request.

### Ethics approval and consent to participate

The study protocol was approved by the ethical committees of the medical faculties of all participating sites: the ethical committees of Berlin (Charite, University Medicine), Bonn, Cologne, Goettingen, Magdeburg, Munich (Ludwig-Maximilians-University), Rostock, and Tuebingen. The process was led and coordinated by the ethical committee of the medical faculty of the University of Bonn. All committees gave ethical approval for this work. All participants provided their written informed consent before inclusion in the study. DELCODE is retrospectively registered at the German Clinical Trials Register (DRKS00007966, 04/05/2015). The DELCODE study was conducted in accordance with the Declaration of Helsinki.

### Consent for publication

Not applicable

### Declaration of generative AI and AI-assisted technologies in the writing process

The authors confirm that no generative AI or AI-assisted technologies were used in the preparation of this manuscript.

Supplementary Figure 1: Batch normalization of plasma GFAP measurements.

The first panel shows raw plasma GFAP concentrations across measurement plates, illustrating inter-plate variability. The second panel displays plasma GFAP values after batch normalization, demonstrating a substantial reduction of plate-related effects while preserving the overall distribution of GFAP levels.

## CRediT authorship contribution statement

**Arda C. Cetindag:** Writing – original draft, Visualization, Validation, Project administration, Methodology, Investigation, Data curation, Conceptualization. **Carola G. Schipke:** Writing – review & editing, Investigation. **Hermann Esselmann:** Writing – review & editing, Investigation. **Niels Kruse:** Writing – review & editing, Investigation. **Jens Wiltfang:** Writing – review & editing, Investigation. **Anja Schneider:** Writing – review & editing, Investigation. **Klaus Fliessbach:** Writing – review & editing, Investigation. **Carolin Miklitz:** Writing – review & editing, Investigation. **Franziska Maier:** Writing – review & editing, Investigation. **Katharina Buerger:** Writing – review & editing, Investigation. **Daniel Janowitz:** Writing – review & editing, Investigation. **Michael Ewers:** Writing – review & editing, Investigation. **Sophia Stöcklein:** Writing – review & editing, Investigation. **Robert Perneczky:** Writing – review & editing, Investigation. **Boris-Stephan Rauchmann:** Writing – review & editing, Investigation. **Carolin Kurz:** Writing – review & editing, Investigation. **Stefan Teipel:** Writing – review & editing, Investigation. **Ingo Kilimann:** Writing – review & editing, Investigation. **Doreen Goerss:** Writing – review & editing, Investigation. **Christoph Laske:** Writing – review & editing, Investigation. **Sebastian Sodenkamp:** Writing – review & editing, Investigation. **Elham Najafpour:** Writing – review & editing, Investigation. **Michael Wagner:** Writing – review & editing, Investigation. **Sandra Roeske:** Writing – review & editing, Investigation. **Ingo Frommann:** Writing – review & editing, Investigation. **Melina Stark:** Writing – review & editing, Investigation. **Frederic Brosseron:** Writing – review & editing, Investigation. **Alfredo Ramirez:** Writing – review & editing, Investigation. **Luca Kleineidam:** Writing – review & editing, Investigation. **Josef Priller:** Writing – review & editing, Investigation. **Eike Jakob Spruth:** Writing – review & editing, Investigation. **Maria Gemenetzi:** Writing – review & editing, Investigation. **Slawek Altenstein:** Writing – review & editing, Investigation. **Emrah Düzel:** Writing – review & editing, Investigation. **Wenzel Glanz:** Writing – review & editing, Investigation. **Enise I. Incesoy:** Writing – review & editing, Investigation. **Michaela Butryn:** Writing – review & editing, Investigation. **Chris Bauer:** Writing – review & editing, Software, Formal analysis, Data curation. **Frank Jessen:** Writing – review & editing, Investigation. **Oliver Peters:** Writing – review & editing, Supervision, Project administration, Methodology, Investigation, Funding acquisition, Conceptualization.

## Declaration of competing interest

The authors declare that they have no known competing financial interests or personal relationships that could have appeared to influence the work reported in this paper.

## Appendix Supplementary materials

Image, application 1

Image, application 2

Image, application 3
