# Supplementary figures and images for: Plasma GFAP outperforms CSF GFAP in detecting amyloid pathology and is associated with increased risk of clinical progression in early Alzheimer’s disease
Source: J Prev Alzheimers Dis. 2026 Mar 28;13(5):100544. doi: 10.1016/j.tjpad.2026.100544 (PMC13054424; doi:10.1016/j.tjpad.2026.100544)

Raw Data

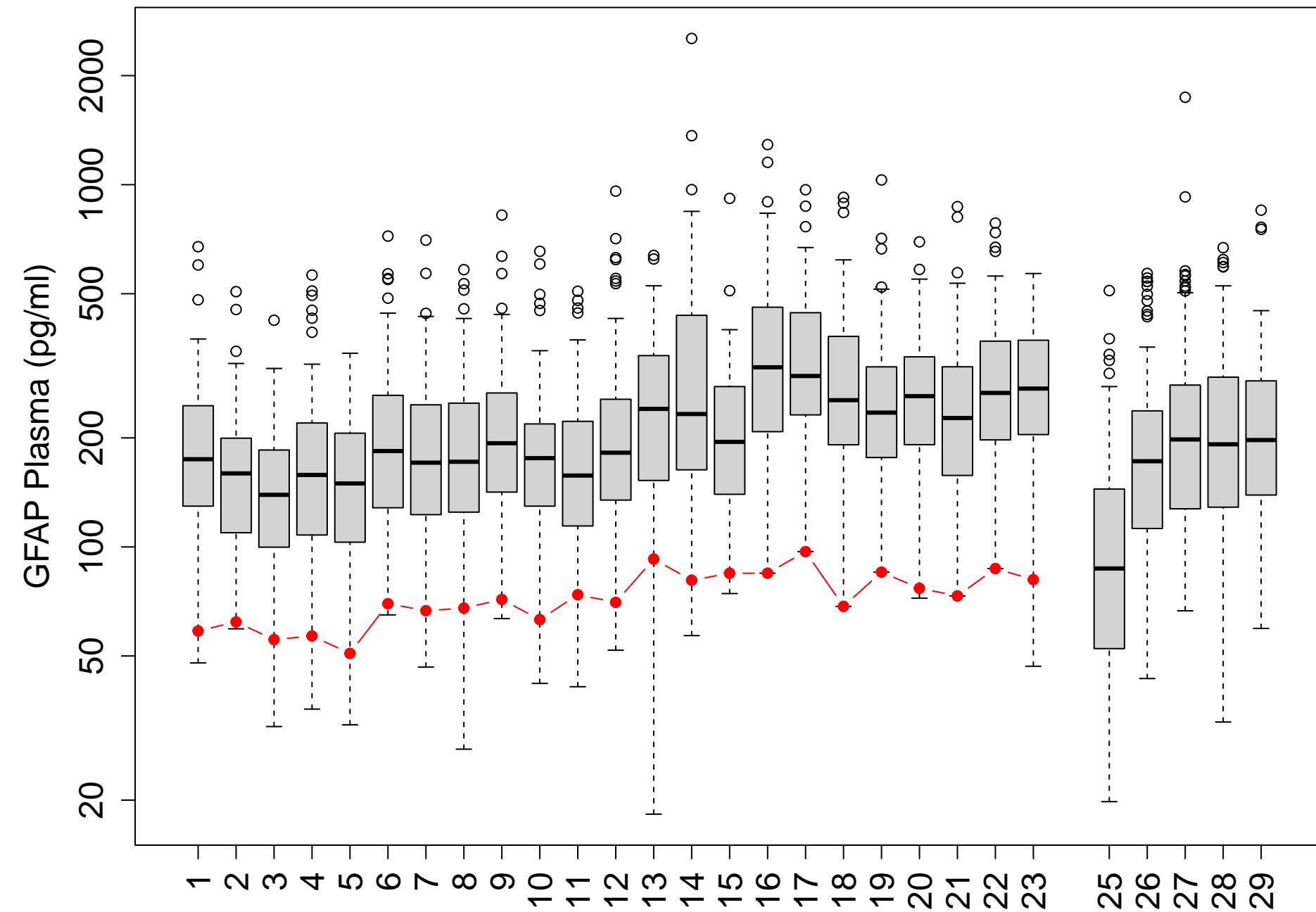

Normalized Data

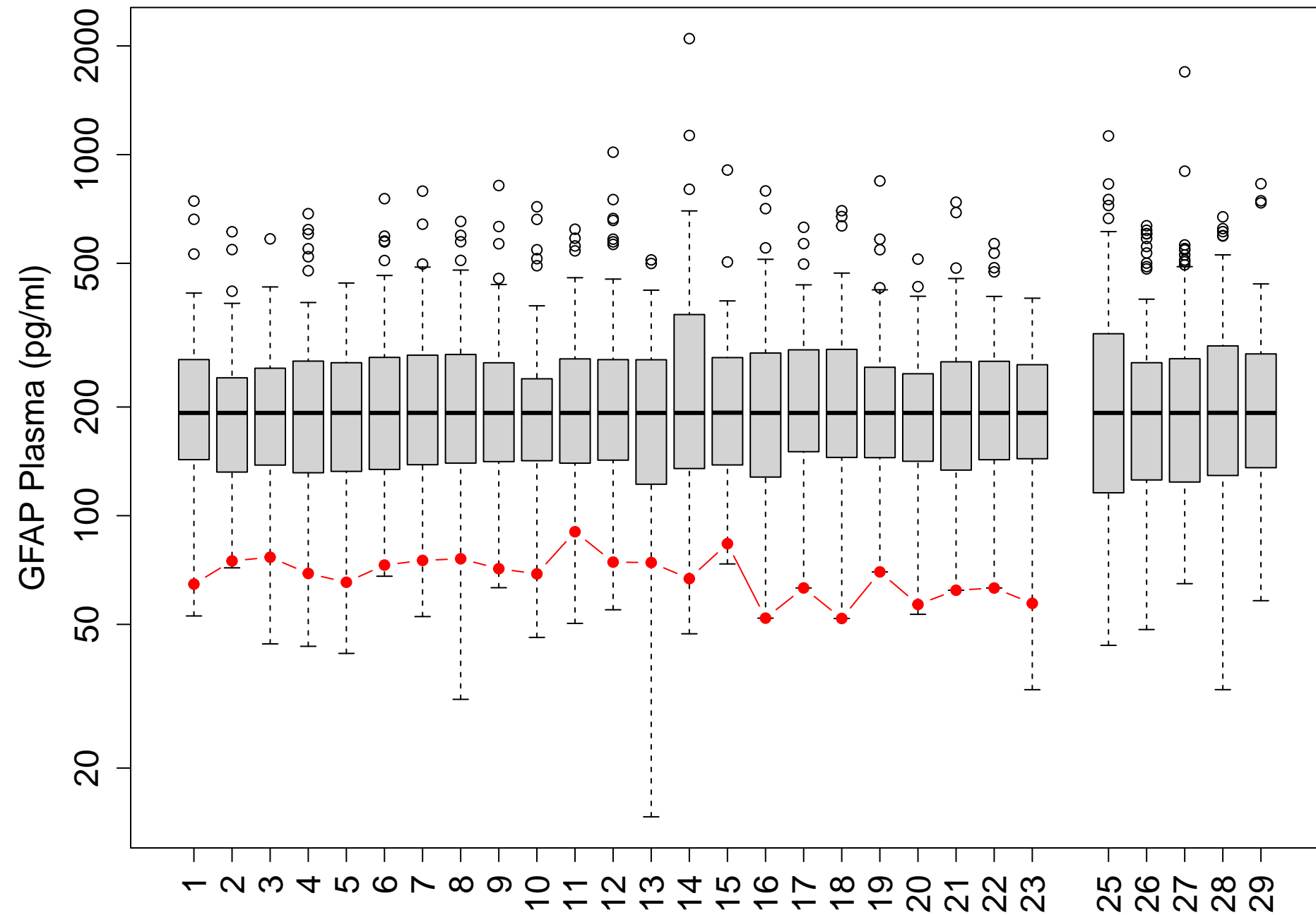

Supplement: Supplementary file 1 [file mmc1.pdf]
