# Supplementary material for: Plasma GFAP outperforms CSF GFAP in detecting amyloid pathology and is associated with increased risk of clinical progression in early Alzheimer’s disease
Source: J Prev Alzheimers Dis. 2026 Mar 28;13(5):100544. doi: 10.1016/j.tjpad.2026.100544 (PMC13054424; doi:10.1016/j.tjpad.2026.100544)
Supplement: Supplementary file 2 [file mmc2.docx]

| Year | n.risk | n.event | n.censor | surv | cumhaz | std.err | std.chaz | lower | upper | Group |
| --- | --- | --- | --- | --- | --- | --- | --- | --- | --- | --- |
| 1 | 412 | 9 | 4 | 0,978812 | 0,02139 | 0,006987 | 0,00713 | 0,965213 | 0,992604 | low |
| 2 | 379 | 13 | 20 | 0,947164 | 0,054216 | 0,010969 | 0,011566 | 0,925908 | 0,968909 | low |
| 3 | 344 | 11 | 24 | 0,918993 | 0,084369 | 0,01354 | 0,014714 | 0,892835 | 0,945917 | low |
| 4 | 289 | 11 | 48 | 0,888027 | 0,118592 | 0,015988 | 0,01798 | 0,857236 | 0,919923 | low |
| 5 | 196 | 12 | 77 | 0,845146 | 0,16798 | 0,019463 | 0,022991 | 0,807848 | 0,884167 | low |
| 1 | 161 | 5 | 3 | 0,970414 | 0,029907 | 0,013034 | 0,013375 | 0,945201 | 0,9963 | high |
| 2 | 135 | 16 | 10 | 0,870128 | 0,138565 | 0,026483 | 0,030315 | 0,819741 | 0,923613 | high |
| 3 | 117 | 10 | 8 | 0,804339 | 0,216761 | 0,03162 | 0,039134 | 0,744692 | 0,868763 | high |
| 4 | 103 | 7 | 8 | 0,755778 | 0,278535 | 0,034633 | 0,045576 | 0,690857 | 0,826799 | high |
| 5 | 66 | 7 | 29 | 0,698572 | 0,3568 | 0,038212 | 0,054399 | 0,627552 | 0,777629 | high |

**Supplementary Table S1. Kaplan–Meier survival statistics for conversion to mild cognitive impairment (MCI) stratified by baseline plasma GFAP levels (low vs. high).**

**Year** indicates years since baseline assessment.
**n.risk** denotes the number of participants at risk at the beginning of each interval.
**n.event** indicates the number of conversions to MCI during the interval.
**n.censor** denotes the number of censored observations.
**surv** represents the Kaplan–Meier estimated conversion-free survival probability.
**cumhaz** indicates the cumulative hazard estimate.
**std.err** and **std.chaz** denote the standard errors of the survival probability and cumulative hazard, respectively.
**lower** and **upper** indicate the 95% confidence interval of the survival estimate.
**Group** indicates plasma GFAP category (low vs. high), based on the Youden-derived threshold (229 pg/ml).
