# Supplementary material for: Plasma GFAP outperforms CSF GFAP in detecting amyloid pathology and is associated with increased risk of clinical progression in early Alzheimer’s disease
Source: J Prev Alzheimers Dis. 2026 Mar 28;13(5):100544. doi: 10.1016/j.tjpad.2026.100544 (PMC13054424; doi:10.1016/j.tjpad.2026.100544)
Supplement: Supplementary file 3 [file mmc3.docx]

| Year | n.risk | n.event | n.censor | surv | cumhaz | std.err | std.chaz | lower | upper | Group |
| --- | --- | --- | --- | --- | --- | --- | --- | --- | --- | --- |
| 1 | 491 | 2 | 13 | 0,996036 | 0,003968 | 0,002798 | 0,002806 | 0,990567 | 1 | low |
| 2 | 455 | 9 | 27 | 0,97735 | 0,022886 | 0,006754 | 0,006904 | 0,964201 | 0,990679 | low |
| 3 | 422 | 3 | 30 | 0,970772 | 0,029632 | 0,007704 | 0,007927 | 0,95579 | 0,985989 | low |
| 4 | 349 | 5 | 72 | 0,958687 | 0,042144 | 0,009319 | 0,00971 | 0,940595 | 0,977127 | low |
| 5 | 245 | 2 | 98 | 0,952441 | 0,048669 | 0,010251 | 0,01075 | 0,932559 | 0,972747 | low |
| 1 | 225 | 1 | 9 | 0,995652 | 0,004348 | 0,004338 | 0,004348 | 0,987185 | 1 | high |
| 2 | 195 | 14 | 16 | 0,931116 | 0,071155 | 0,017177 | 0,018392 | 0,898051 | 0,965399 | high |
| 3 | 172 | 9 | 14 | 0,886875 | 0,119704 | 0,021794 | 0,024503 | 0,845172 | 0,930636 | high |
| 4 | 146 | 7 | 20 | 0,849373 | 0,162741 | 0,025069 | 0,029421 | 0,801632 | 0,899957 | high |
| 5 | 98 | 3 | 44 | 0,828968 | 0,186959 | 0,027122 | 0,032608 | 0,777478 | 0,883868 | high |

Supplementary Table S2. Kaplan–Meier survival statistics for conversion to Alzheimer’s disease dementia stratified by baseline plasma GFAP levels (low vs. high).

**Year** indicates years since baseline assessment.
**n.risk** denotes the number of participants at risk at the beginning of each time interval.
**n.event** indicates the number of conversions to Alzheimer’s disease dementia during the interval.
**n.censor** denotes the number of censored observations.
**surv** represents the Kaplan–Meier estimated dementia-free survival probability.
**cumhaz** indicates the cumulative hazard estimate.
**std.err** and **std.chaz** denote the standard errors of the survival probability and cumulative hazard, respectively.
**lower** and **upper** indicate the 95% confidence interval of the survival estimate.
**Group** indicates plasma GFAP category (low vs. high), based on the Youden-derived threshold of 229 pg/ml.
